# Assessment of chronic disease management mode (CDMM) on participants with primary hypertension

**DOI:** 10.1111/tmi.13577

**Published:** 2021-05-06

**Authors:** Dan Ling, Rong Wang, Qun Chen, Xinyuan Liu, Xueli Qi, Chen Chen, Xiaoman Shi, Zhaoheng Dong

**Affiliations:** ^1^ Department of Cardiology Second Affiliated Hospital of Xi'an Jiaotong University Xi'an City China; ^2^ Key Laboratory of Trace Elements and Endemic Diseases Institute of Endemic Diseases Xi'an Jiaotong University Health Science Center Xi’an China; ^3^ Endocrinology Faculty of Medicine School of Biomedical Sciences University of Queensland St Lucia Qld Australia; ^4^ Shandong Shenghua Electronic New Materials Co., Ltd. Laiyang City China

**Keywords:** chronic disease management mode, systolic blood pressure, QLICD‐HY, primary hypertension, nurse

## Abstract

**Objective:**

Hypertension requires continuous and long‐term care to prevent associated complications. Chronic disease management mode (CDMM) was developed to improve patients' self‐management. We aimed to evaluate quality of care and clinical outcomes of CDMM versus routine care.

**Methods:**

300 patients aged >30 years with primary hypertension were randomly allocated to the CDMM intervention group (*n* = 162) or the usual care control group (*n* = 138). CDMM comprised nursing consultations, telephone contact, online WeChat link, health education, and appropriate referrals during hospitalisation and after discharge. QLICD‐HY (V 2.0) scale was used to evaluate the quality of life. Care outcomes were biochemical parameters, body mass index, blood pressure levels, waist circumference, psychological indicators and quality of life assessed on admission (baseline) and one month post‐care for both groups. Data were collected and analysed using SPSS 20.0.

**Results:**

After one month, the intervention group had 6 mm Hg (95% CI: −5.12 to −9.08) lower SBP and 0.6 mM/L (95% CI: −0.52 to −0.68) lower LDL than the control group. In terms of improvements in BMI, UmAlb or waist circumference, there were no differences between both groups. The intervention group scored better on psychological indicators than controls (*P* < 0.05), and scores reflecting social and psychological function in the intervention group were significantly higher than scores at baseline, and higher than scores of controls after one month (*P* < 0.05). In the control group, there was no statistically significant difference between the scores at baseline and after one month.

**Conclusions:**

Under CDMM hypertension care, improvement of blood pressure and LDL was clinically significant. Intervention care further improves social and psychological function among participants with primary hypertension.

## Introduction

Cardiovascular diseases have emerged as the leading cause of death globally [1]; they were responsible for 17.8 million deaths in 2017 [2]. Hypertension, one of many biomedical risk factors from cardiovascular disease, causes more deaths than any other single factor [3]. A total of 1.38 billion people worldwide are estimated to have hypertension without optimal control [4].

In 2018, there were more than 200 million people with hypertension in China, and the number was rising rapidly [5]. Failures in the management of hypertension contribute to severe complications, compromised quality of life and a significant economic burden [6]. Uncontrolled hypertension is a great risk of cerebrovascular and cardiovascular stroke [7]. Hyperglycaemia, hyperlipidemia, obesity, smoking and drinking are the modifiable risk factors for blood pressure control [8]. Reducing high blood pressure may reduce the morbidity and mortality from cardiovascular disease [9]. As lifestyle changes may prevent the occurrence of hypertension, improving the control of blood pressure in chronic disease management is a necessity.

A scheme called chronic disease management mode (CDMM) has been adopted to improve health outcomes and to reduce long‐term costs by restructuring health systems in a multidimensional manner [10]. CDMM has not been widely adopted in hospitals, due partially to their weak effectiveness in randomised control trials and concerns on acceptance by patients [11]. We designed and implemented a CDMM that included new standard measurements (e.g. depression screening, care planning, health coaching) to support self‐management of hypertension. Although models of CDMM vary internationally, it often represents optimal hypertension management [12]. Despite the availability of effective pharmacotherapy, self‐management support, hypertension knowledge, self‐confidence and behaviour control are closely associated with enhanced antihypertensive effects [13]. CDMM emphasises patient self‐management after intervention. This study aimed to analyse whether this particular CDMM improved clinical outcomes of primary hypertension.

## Methods

### *Study* *design*

A total of 300 primary hypertension inpatients were enrolled from March 2016 to December 2018 in the Department of Cardiovascular Medicine, the Second Affiliated Hospital of Xi'an Jiaotong University. Participants were allocated to the CDMM intervention group or the usual care control group using the random number table method. Inclusion and exclusion criteria in the ‘Integrated Assessment Form for Primary Hypertension Participants’ were used to assess participants' eligibility for the study. ‘Integrated Assessment Form for primary hypertension participants’, ‘Health Management Assessment Form for primary hypertension participants’, ‘Psychological Assessment Form’ and ‘Health Management Execution Form for primary hypertension participants’ were applied to participants. Quality of life for hypertensive participants was evaluated by QLICD‐HY scoring.

The investigating team consisted of two trained nurses, one head nurse, one researcher and two coordinators. Nurses were in charge of follow‐ups, psychological interviews and health education. The researcher was in charge of support for the project execution and assessment of the quality of care. The coordinators were responsible for data entry and programme maintenance.

All participants were diagnosed as suffering from primary hypertension. Subjects whose parents had hypertension were considered to have a positive family history, which was recorded in this study.

### *Inclusion* *and exclusion criteria*

Inclusion criteria were a diagnosis of primary hypertension, with a clear history of hypertension and age >30 years (2010 Chinese guidelines for the management of hypertension). Blood pressure was measured three times on different days. The systolic blood pressure (SBP) was ≥140 mmHg and/or DBP ≥ 90 mmHg. All participants gave written informed consent.

Exclusion criteria were ① secondary hypertension; ② inability to participate in the experiment due to physical disability, mental illness or cognitive functional difficulties; ③pregnancy or breastfeeding; and ④chronic heart failure symptoms, liver damage, tuberculosis, severe anaemia, thyroid disease or incomplete personal data.

The aetiology of primary hypertension is unclear, but it seems to be caused by decompensation of the regulatory mechanism of normal blood pressure, probably due to multiple environmental factors interacting with genetic factors. Non‐primary hypertension refers to hypertension caused by specific identified diseases or causes.

### *Consultations* *by nurses*

The ‘Health Management Assessment Form for primary hypertension participants’ was used to record relevant knowledge and existing main problems of participants, including the basic health, disease‐related, drug‐related and lifestyle‐related knowledge, plus individual‐level information needs. The data were collected via nursing consultations lasting approximately 40 min on admission (baseline) and at the 1‐month follow‐up.

According to the evaluation results, personalised plans for participants in the intervention group were developed, including the time, place, content, method and goal of the intervention. Details were documented in the ‘Health Management Execution Form for primary hypertension participants’. Blood pressure, BMI and waist circumference were followed up 4 times a year. Control group participants received routine nursing care.

### *Psychological* *intervention by nurses*

On admission day, the ‘Psychological Assessment Form’ was used to evaluate the psychological status of all participants. Patients were screened for indicators of depression, irritability, mania, anxiety, somatisation, psychosis, suicide, memory impairment, obsessive compulsive disorder, life pressure, type A behaviour etc. These indicators may have a negative effect on the outcome of hypertension treatment. Screening was based on advice by the doctors in the team and done by a nurse through a 15–30‐min psychological interview. The nurse provided psychological counselling on these targeted positive indicators.

### *Interventions* *by nurses during hospitalisation*

Based on the results of ‘Health Management Assessment Sheet for participants with primary hypertension’, the nurse‐in‐charge administered appropriate intervention measures. Specifically, from the day of admission to the day of discharge, patients underwent daily health education focused on self‐managing changeable risk factors. The patient thereafter could simply repeat the process without a nurse. If not, education was repeated, including risk factors that required special attention. One day before discharge, a 15–30‐min psychological interview was conducted with intervention participants who had screened positive for indicators of psychological illness. Controls received standard care comprising blood pressure monitoring, and medical and nursing appointments.

### *Intervention* *activities by nurses after discharge*

Post‐discharge, WeChat and telephone contact were used to communicate. We established a WeChat official account and a WeChat group for participants with primary hypertension and uploaded hypertension‐related knowledge to the WeChat group and WeChat official account, such as the characteristics of hypertension, the main hazards of the disease, dietary advice, exercise guidance, help sleep and improve drug compliance. Questions from participants were answered one‐to‐one by experts through WeChat. WeChat group and WeChat official accounts were jointly managed by the head nurse and the researcher. For complex cases, doctors and nurses took the form of individual cases for a group discussion and targeted answers were conveyed to the participants. If necessary, participants were invited to the follow‐up centre for consultation.

### *Intervention* *activities*

Intervention activities included sending lecture information online and offline focusing on topics such as developing a healthier lifestyle (by exercising more, eating less salt and more fresh fruit and vegetables, stopping smoking and drinking), regular exercise, maintaining effective treatment, regularly taking blood pressure and prevention of chronic complications of hypertension. Lectures were held once a week by the nurses in charge. The information of the lectures was distributed via WeChat group and WeChat public account, and participants were invited to attend. Individualised educational activities were provided by the nurse during consultations through WeChat and telephone calls.

### Follow‐up

The follow‐up visit was scheduled 1‐month post‐discharge. It lasted approximately 40 min and included tailored health education, blood pressure measurement, waist circumference measurement and body mass index (BMI) calculation. Health guidance was given to intervention participants with indicators of psychological illness. Participants who were unable to follow‐up on time were called, and their knowledge of hypertension, lifestyle changes and physiological indicators was assessed.

### *Outcome* *measures*

Full data were collected at the beginning of the study (T0) and at the 1‐month follow‐up (T1) for both intervention and control groups. The assessment was conducted at the beginning (T0) for both groups. Participants were asked to again complete the ‘Health Management Assessment Form for primary hypertension participants’ at follow‐up to evaluate the improvement of knowledge and main problems. Blood pressure and BMI, waist circumference (WC), psychological indicators and quality of life were observed. Blood pressure was measured and classified in accordance with the 2010 Chinese guidelines for the management of hypertension [14], that is taken with a professional blood pressure monitor three times on the right arm after the patient had been sitting comfortably for 5 min.

Urine microalbumin (UmAlb) was analysed by transmission turbidimetric method using urine analyser from Beckman Coulter Company. Fasting blood glucose (FBG) was measured by Hitachi 7600‐100 (Japan No.1 Chemical Co., Ltd). Low‐density lipoprotein was determined by commercial kits. For weight, and height and waist circumference (WC) measurement participants had to take off their shoes and wear a single layer of clothes.

### *Quality* *of life assessment*

Quality of life was assessed with QLICD‐HY (V 2.0) developed by Wan et al. [15] upon admission and one month after discharge. Height and weight were measured by a standard device. BMI of each patient was calculated as the weight (kilograms) divided by the squared height in metres. The questionnaire consisted of 47 items. Responses were rated on a 5‐point scale ranging from 0 (not at all) to 5 (very much) and higher scores indicated better quality of life.

### *Statistical* *analysis*

Baseline data were compared using chi‐squared tests for categorical variables by the SPSS Statistics version 20.0 (IBM Corp. Armonk, NY). Two‐tailed Student’s *t*‐tests and ANOVA were employed to determine the difference for numerical variables. The risk factors age, sex, years of education, smoking, drinking, career, average annual income, family history of hypertension, course of the disease and grade of hypertension were analysed by chi‐squared tests. 95% confidence intervals (CI) for all the parameters were estimated. The *P* < 0.05 was considered statistically significant.

### Ethical approval

Ethical approval was obtained from Ethical Committee of Xi’an Medical University Health Science Center (Licence number 2016056).

## Results

As shown in Table 1, there was no significant difference between groups in terms of sociodemographic and risk factors.

**Table 1:** Baseline sociodemographic and risk factors of the participants

Variables	*N* = 300	Intervention care Group	Usual care group	*P* value
		*N* = 162	*N* = 138	
Sex, *n* (%)				
Male	210 (70.00)	113 (69.75)	97 (70.29)	0.91
Female	90 (30.00)	49 (30.25)	41 (29.71)	
Age				
35–50	14 (4.67)	7 (4.32)	7 (5.07)	0.94
50–65	136 (45.33)	73 (45.06)	63 (45.65)	
≥65	150 (50.00)	82 (50.62)	68 (49.28)	
Years of education				
<9	145 (48.33)	78 (48.15)	67 (48.55)	0.962
9–12	128 (42.67)	70 (43.21)	58 (42.03)	
13–16	27 (9.00)	14 (8.64)	13 (9.42)	
Career				
White‐collar worker	69 (23.00)	37 (22.84)	32 (23.19)	1.00
Blue‐collar worker	85 (28.33)	46 (28.40)	39 (28.26)	
Retired	85 (28.33)	46 (28.40)	39 (28.26)	
Freelance or unemployed	61 (20.34)	33 (20.37)	28 (20.29)	
Average annual income(RMB/year)				
<3500	51 (17.00)	28 (17.28)	23 (16.67)	0.95
3500–6000	102 (34.00)	56 (34.57)	46 (33.33)	
>6000	147 (49.00)	78 (48.15)	69 (50.00)	
Home address				
City and town	195 (65.00)	106 (65.43)	89 (64.50)	0.865
Village	105 (35.00)	56 (34.57)	49 (35.51)	
Grade of hypertension, *n* (%)				
Grade I hypertension (SBP 140–159 or/and DBP 90–99 mmHg)	10 (3.33)	5 (3.09)	5 (3.62)	0.967
Grade II hypertension (SBP 160–179 or/and DBP 100–109 mmHg)	85 (28.33)	46 (28.40)	39 (28.26)	
Grade III hypertension (SBP ≥ 180 or/and DBP ≥ 110 mmHg)	205 (68.34)	111 (68.52)	94 (68.12)	
Smoking				
Yes	95 (31.67)	51 (31.48)	44 (31.88)	0.94
No	205 (68.33)	111 (68.52)	94 (68.12)	
Drinking				
Yes	42 (14.00)	23 (14.20)	19 (13.77)	0.915
No	258 (86.00)	139 (85.80)	119 (86.23)	
Family history of hypertension				
Yes	124 (41.33)	74 (45.68)	50 (36.23)	0.098
No	176 (58.67)	88 (54.32)	88 (63.77)	
Course of the disease				
1–10 year	45 (15.00)	23 (14.20)	22 (15.94)	0.683
10–20 year	99 (33.00)	51 (31.48)	48 (34.78)	
≥20 year	156 (52.00)	88 (54.32)	68 (49.28)	

### *Awareness* *rate of hypertension knowledge and lifestyle improvement*

**Figure 1:**
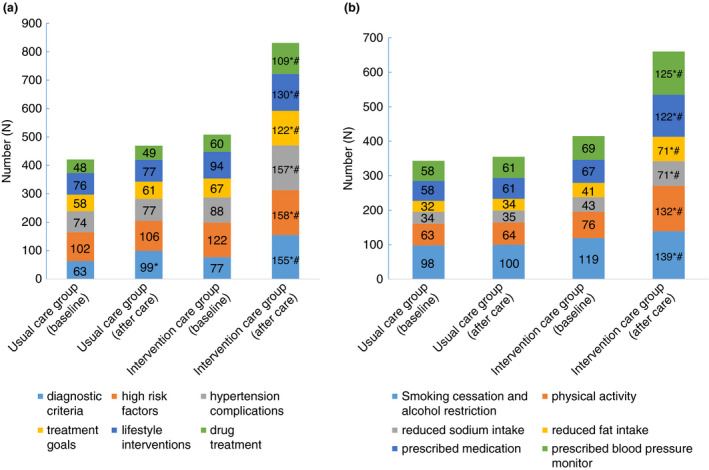
Awareness rate of hypertension knowledge and the number of participants for lifestyle changing at baseline and after care. (a) The number of participants who knew the diagnostic criteria, high risk factors, hypertension complications, treatment goals, lifestyle improvements and drug treatment of hypertension were shown in two groups at baseline and after care. (b) The number of participants who took smoking cessation and alcohol restriction, reduced fat intake, regular physical activity, reduced sodium intake, decreased oil intake, prescribed medication and prescribed blood pressure monitor were shown in two groups at baseline and after care. *P < 0.05 between one‐month care and baseline; #*P* < 0.05 represents statistically significant difference when compared with the Usual care group after care.

There was no significant difference in the knowledge about hypertension between the two groups at baseline (*P* > 0.05; Figure 1a). After one month, the number of participants who knew the diagnostic criteria, high‐risk factors, complications, treatment goals, lifestyle improvements and drug treatment of hypertension was significantly larger in the intervention group than at baseline (*P* < 0.05) and also larger than the post‐intervention level of the control group (*P* < 0.05). The number of controls knowing the diagnostic criteria for hypertension after one month was significantly larger than at baseline (*P* < 0.05). There were no significant changes in any of the other indicators.

Participants from the intervention group ceased smoking, reduced drinking, increased exercise, reduced sodium intake, decreased oil intake and maintained medications. All indices were better than in the control group (*P* < 0.05) and also significantly better than that at baseline. In controls, by contrast, indicators did not differ significantly between baseline and follow‐up (*P* > 0.05; Figure 1b).

### *Blood* *pressure and physiological index improvement*

**Figure 2:**
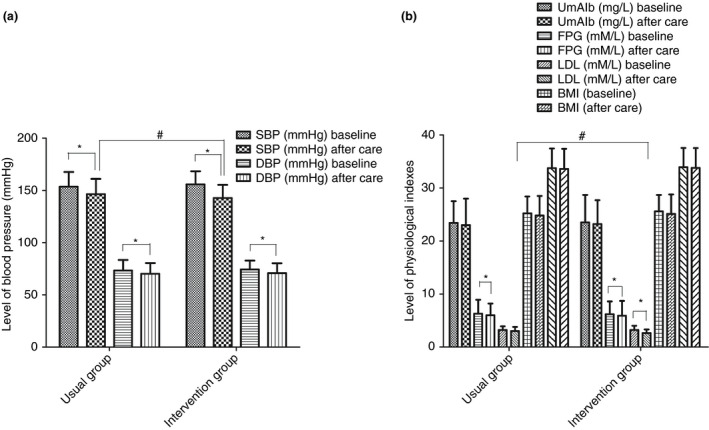
The SBP, DBP, UmAlb, FPG, LDL, BMI and WC for two groups at baseline and after one month. Data were shown as mean ± S.D., *N* = 162 and 138 for intervention care group and the usual care group, respectively. *P < 0.05 represented statistically significant difference between after care and that at baseline. # *P* < 0.05 represented statistically significant difference between intervention group after care and usual group after care.

At follow‐up, SBP and DBP of both groups were significantly lower than at baseline, but the SBP of the intervention group was 6 mm Hg (95% CI: −5.12 to −9.08) lower than that of controls (*P* < 0.05; Figure 2a). FBG levels in both groups were significantly lower at follow‐up than at baseline (*P* < 0.05). In the intervention group, LDL after one month was 0.6 mM/L (95% CI: −0.52 to −0.68) lower than in controls (*P* < 0.05). There was no significant difference between the two groups in terms of BMI, waist circumference (WC) and UmAlb between baseline and follow‐up (*P* > 0.05; Figure 2b).

### *Psychological* *indicator improvement*

Although there were no significant differences in psychological indicators between the two groups at baseline (*P* > 0.05), scores for depression, anxiety, life events and total mental symptoms, and scores for social activities in the intervention group had significantly improved at follow‐up and were also significantly better than those of controls (*P* < 0.05). The score for irritability in the intervention care group had dropped significantly since baseline. In the control group, the scores for anxiety and total mental symptoms had also significantly decreased from baseline (Table 2).

**Table 2:** Psychological indicators of the participants after care ($\bar{X}$±S.D.)

Dimension	Item	Intervention care group (*N* = 162, baseline)	Intervention care group (*N* = 162, after care)	*P* value	Usual care group (*N* = 138, baseline)	Usual care group (*N* = 138, after care)	*P* value
Mental symptoms	Depression	1.52 ± 0.83	1.27 ± 0.51*	0.004	1.61 ± 0.84	1.52 ± 0.64	0.811
	Irritability	1.60 ± 0.72	1.49 ± 0.72	0.000	1.61 ± 0.89	1.59 ± 0.82	0.611
	Manic	1.04 ± 0.17	1.02 ± 0.12	0.070	1.02 ± 0.10	1.01 ± 0.07	0.590
	Anxiety	1.48 ± 0.55	1.22 ± 0.29*	0.000	1.54 ± 0.63	1.40 ± 0.48	0.009
	Somatisation	1.34 ± 0.68	1.32 ± 0.66	0.905	1.43 ± 0.67	1.41 ± 0.64	0.428
	Psychotic	1.03 ± 0.26	1.02 ± 0.15	0.325	1.02 ± 0.16	1.01 ± 0.27	0.670
	Suicide	1.07 ± 0.33	1.03 ± 0.15	0.180	1.05 ± 0.27	1.08 ± 0.34	0.274
	Memory impairment	1.53 ± 0.81	1.48 ± 0.76	0.158	1.59 ± 0.90	1.58 ± 0.89	0.759
	Obsessive compulsive disorder	1.12 ± 0.28	1.06 ± 0.29	0.738	1.16 ± 0.38	1.10 ± 0.33	0.206
	Total score of mental symptoms	12.17 ± 2.85	10.80 ± 1.59*	0.000	12.27 ± 2.55	11.70 ± 2.09	0.012
Social situation	Interpersonal relationships	2.42 ± 0.77	2.34 ± 0.79	0.449	2.43 ± 0.66	2.42 ± 0.63	0.995
	Life pressure	2.28 ± 0.83	2.23 ± 0.79	0.808	2.07 ± 0.89	2.42 ± 0.63	0.385
	Life events	2.08 ± 0.95	1.23 ± 1.02*	0.000	2.14 ± 0.97	2.24 ± 1.11	0.968
	Total score of Social situation	6.97 ± 2.00	6.81 ± 2.02*	0.005	6.81 ± 1.72	6.79 ± 1.68	0.856
Personality (personality)	Time urgency	3.75 ± 0.77	3.71 ± 0.77	0.626	3.61 ± 0.81	3.58 ± 0.73	0.818
	Competitive	3.44 ± 0.87	3.43 ± 0.86	0.396	3.43 ± 0.77	3.41 ± 0.73	0.261
	Type A behaviour	7.16 ± 1.49	7.14 ± 1.46	0.959	6.43 ± 1.89	7.00 ± 1.32	0.712

* Represents when compared with the Usual care group after care *P* < 0.05.

### *Quality* *of life improvement*

At follow‐up, the scores of mean scales (mean of the sum of the total scores of the four modules) of social function and psychological function of the intervention group were significantly higher than at baseline and significantly higher than those of controls at follow‐up (*P* < 0.05). In the control group, there was no statistically significant difference before and after conventional care (*P* > 0.05; Table 3).

**Table 3:** Mean improvement of quality of life in two groups after care ($\bar{X}$±S.D.)

Domain	Intervention care group (*N* = 162)	Usual care group (*N* = 138)	*P* value
Mean score of scale	4.24 ± 2.12^†^	0.74 ± 3.25	0.000
Specific module	0.59 ± 1.58	0.72 ± 2.02	0.059
Social function	3.04 ± 2.19^†^	0.69 ± 2.08	0.000
Psychological function	2.14 ± 2.09^†^	0.61 ± 2.04	0.000
Physiological function	0.63 ± 1.54	0.68 ± 2.07	0.278

The results were calculated by the score (after care)‐the score (baseline).

† Represents when compared with that at baseline *P* < 0.05. The specific module is hypertension specific module. It contains items such as symptoms of hypertension, side effects of drugs and effects on psychological life.

## Discussion

In this study, we assessed the effect of a nurse‐led WeChat platform to aid discharged primary hypertension patients in managing their condition. WeChat is China's most popular social mobile app with more than 1.13 billion active users [16]. Due to its flexible and multiple functions, WeChat is a cost‐effective tool for hypertension self‐management [17].

Health knowledge can be rapidly disseminated through the powerful applications of WeChat. One survey found that 71.4% of participants received their health education through websites such as Baidu and WeChat [18]. Low awareness of hypertension is a significant barrier to its control, especially in Northwest China, including Xi’an in Shaanxi Province, China [19]. At the beginning of the programme, knowledge about hypertension was very poor, so nurses educated their patients while they were hospitalised. After discharge, nurses related knowledge, such as diagnostic criteria for hypertension, disease hazards, dietary advice, physical exercise and sleep promotion through a WeChat group and a WeChat public account. Health education through CDMM improved the hypertension knowledge of participants such that they became more confident in controlling their blood pressure and motivated to change some behaviours. This resulted in improved awareness of diagnostic criteria, high‐risk factors, complications, treatment goals, lifestyle improvements, and drug treatment of hypertension.

CDMM was established in our study and enhanced the beneficial hypotensive effects. A decrease of more than 4.6 mmHg in SBP and 2.2 mmHg in DBP was considered to be sufficient to prevent cardiovascular events, and in this study, the reduction was 13.1 mmHg for SBP and 3.4 mmHg for DBP [20]. Our findings confirm those of a previous study that people with hypertension managed with CDMM showed a significant reduction in SBP and DBP [21].

Age, gender, BMI, waist circumference, smoking and family history are well‐known risk factors for hypertension [22]. Among these, age, gender and family history are unchangeable, whereas BMI, waist circumference and smoking are changeable and can be improved by lifestyle changes and physical exercise. In our intervention group, BMI, waist circumference and smoking were improved, but not to a statistically significant degree, perhaps because the follow‐up period of 1 month was too short.

Fasting glucose and LDL levels in patients with primary hypertension are higher than in healthy people [23]. Through CDMM fasting blood glucose and LDL decreased significantly from baseline and ended up lower than in controls at follow‐up. This indicates that awareness and lifestyle improvements prompted by CDMM effectively reduce fasting blood glucose and LDL.

The ‘Psychological Assessment Form’ in DSM‐5 is a tool for definitive diagnosis of psychiatric disorders [24]. As measured by this form, anxiety and depression are highly prevalent among hypertensive participants [25, 26]. Reduced depression, irritability and anxiety in our intervention group indicated that participants became more confident in managing their emotions.

Quality of life (QOL) assessment is an important indicator of health status and the effectiveness of intervention measures. The Short Form Health Survey (SF‐36) is widely used to evaluate QOL of hypertension [27], but symptoms and side effects specific to hypertension are not included. Quality of Life Instruments for Chronic Diseases (QLICD) were developed and validated by Wan [15] by combining the general module (QLICD‐GM) with the specific module for hypertension. QLICD‐HY (V 2.0) is an improved version that has not yet been widely used nationwide. It seems that nurse consultation, interview and health education may impact positively on most dimensions of QLICD‐HY in participants with primary hypertension. Patient education using WeChat improved the knowledge of hypertension and overall quality of life [28] very effectively. Participants with higher self‐management skills developed better blood pressure control, diet management and emotion management (Table 4).

**Table 4:** Summary of all the surveys used in the study

Surveys	Key assessment
Integrated Assessment Form for primary hypertension participants	Whether to enter the study or not
Health Management Assessment Form for primary hypertension participants	Risk factor assessment
Psychological Assessment Form	Mental stress, mental health status assessment
Health Management Execution Form for primary hypertension participants	Health education and promotion
QLICD‐HY(V2.0)	Quality of life assessment

Our study had limitations. Nurse‐led CDMM was tested in participants from the intervention and control group because the intervention strategies had not been completely blinded to participants and nurses. Further studies are warranted to avoid this limitation. The nurses focused on health promotion, psychological interview and maintenance aspects. Psychological interviews may require specialised knowledge of psychology. Nurses only had a short‐term training, and the interview effect might be difficult to judge.

The nurses' traditional role in hypertension management was expanded from hospital to outside, and to more important roles, such as assessment and consultations. This nurse‐based CDMM could be implemented for other chronic diseases.

## Conclusion

CDMM applied to adult inpatients with primary hypertension had a significant effect, mainly showing reduction in SBP and LDL, and significant improvement in knowledge on hypertension, lifestyle, depression, anxiety, life events and quality of life. WeChat is an effective tool for health education of hypertension.
